# Comparative evaluation of polymerase chain reaction assay with microscopy for detection of asymptomatic carrier state of theileriosis in a herd of crossbred cattle

**DOI:** 10.14202/vetworld.2016.1039-1042

**Published:** 2016-09-30

**Authors:** Gaurav Charaya, N. K. Rakha, Sushila Maan, Aman Kumar, Tarun Kumar, Ricky Jhambh

**Affiliations:** 1 Department of Veterinary Medicine, Lala Lajpat Rai University of Veterinary and Animal Sciences, Hisar, Haryana, India; 2 Department of Animal Biotechnology, Lala Lajpat Rai University of Veterinary and Animal Sciences, Hisar, Haryana, India; 3 Teaching Veterinary Clinical Complex, Lala Lajpat Rai University of Veterinary and Animal Sciences, Hisar, Haryana, India

**Keywords:** Carrier state, *cytochrome b*, Giemsa staining, polymerase chain reaction assay, theileriosis, *Theileria annulata*

## Abstract

**Aim:**

This study aims to develop and to standardize a polymerase chain reaction (PCR) assay that will diagnose clinical as well as carrier state of the disease and to compare the results with conventional microscopy technique.

**Materials and Methods:**

A herd of crossbred cattle with the previous history of theileriosis in village Lahli, district Rohtak, Haryana, was selected for this study. A total of 29 blood samples were collected randomly from cows including five clinically ill cattle. Blood smears from all animals and lymph node biopsy smears from animal with swollen lymph nodes were examined microscopically after conventional Giemsa staining. Phenol chloroform isoamyl alcohol method was used for extracting DNA from blood. Previously published primers targeting *cytochrome b* gene sequence of *Theileria annulata* were used in the PCR assay that was standardized to use in the laboratory.

**Results:**

Out of 29 samples tested,18 (62.06%) were found positive for theileriosis by PCR assay, whereas only 10 (34.48%) samples were detected positive by conventional microscopic technique using Giemsa staining method.

**Conclusions:**

On the basis results of comparative studies, it can be concluded that PCR assay is a more sensitive than microscopic examination for detection of theileriosis. This can be attributed to the ability of PCR assay to detect small amounts of genomic DNA of *T. annulata* or low parasitemia in cows. Therefore, PCR assay can serve as a more sensitive tool to detect *Theileria* for detection of theileriosis even in asymptomatic carrier cattle which is important for the implementation of successful control programs.

## Introduction

In India, bovine tropical theileriosis is an important endemic hemoprotozoan disease caused by *Theileria annulata*. The disease is characterized by a marked rise in body temperature, reaching 40-41.5°C, depression, lacrimation, nasal discharge, swelling of the superficial lymph nodes, and anemia. It is transmitted by *Hyalomma anatolicum*, a three host tick which act as a biological vector for *T. annulata*. Recovered cows from acute or primary theileriosis, remain infected for a long period and even for the rest of their lives, acting as reservoirs of infection for ticks which cause natural transmission of the disease [1,2].

The conventional method for identification of organism includes an examination of blood smears using Giemsa staining. Indeed, Giemsa-stained blood smears can be used as a suitable method to detect *Theileria* in the animals suspected to be suffering from clinically acute theileriosis, but it is not applicable for the detection of presymptomatic or carrier animals, where parasitemia is very low [3]. Identification of such animals is, therefore, crucial for the accurate assessment of epidemiology of the disease and the implementation of control programs aimed at improving productivity.

Molecular methods, with a high degree of sensitivity and specificity, have been developed to identify various *Theileria* species in persistently infected cattle [4]. Therefore, this study was planned to standardize the polymerase chain reaction (PCR) assay for detection of *T. annulata* – infected asymptomatic carrier animals and to compare PCR assay with microscopic examination.

## Materials and Methods

### Ethical approval

Samples were collected by qualified veterinarians as per standard sample collection method without any stress/harm to animals and with due permission of the Institutional Animal Ethics Committee.

### Sample collection

Crossbred cattle herd with a recent history of three clinical cases of theileriosis detected microscopically was selected for sampling. A total of 29 blood samples (10% approximately) from a herd of 250 cattle comprising 67 calves and 183 adult animals were collected by random sampling including five clinically ill cattle in vials containing ethylene diamine tetra acetic acid. The clinically affected animal was having a fever, pale mucous membrane, and swollen lymph nodes. Thin blood smears were prepared from each sample at the site of sample collection and fixed using methanol and remaining part of the sample was brought to the laboratory for DNA extraction. Lymph node biopsy was also taken from four animals having swollen pre-scapular lymph nodes, and a smear was made and fixed in methanol at the site of sample collection.

### Clinical observations and history taking

Age, sex, lymph node examination, mucous membrane examination, and rectal temperature of all 29 animals included in the study were recorded.

### Microscopic examination

Fixed thin blood smear and lymph node biopsy smears were stained with Giemsa stain for 30 min [5]. Blood smears were examined for intra erythrocytic forms (signet ring, dot, orcomma shaped) of *T. annulata* piroplasm under 100×objective magnifications. About 20 microscopic fields, per slide, were observed to view the parasite. The presence of single piroplasms was recorded as positive for *T. annulata*. Fixed lymph node biopsy smears were examined under 100×magnifications in the search for characteristic Koch blue bodies, the presence of which is confirmatory diagnosis of theileriosis.

### DNA extraction

DNA was extracted using phenol-chloroform isoamyl alcohol method. To 500 μl of whole blood three volume of red blood cell lysis buffer was added and kept for 15 min at 37°C. To this 600 μl of DNA extraction buffer was added followed by 10% sodium dodecyl sulfate (260 μl). Proteinase K (20 μl) was added and the sample was incubated at 50°C for 1h. After 1 h of incubation, an equal volume of saturated phenol was added and kept for 15 min at 37°C. Centrifugation at 12,000 rpm was done and upper aqueous phase was separated. To aqueous phase, the equal volume of phenol-chloroform isoamyl alcohol was added and centrifuged at 12000 rpm for 15 min. Upper aqueous phase was separated in a fresh Eppendorf and to it, chloroform isoamyl alcohol was added in equal volume, mixed and centrifuged at 12,000 rpm for 15 min. To aqueous phase 1/10^th^ volume of 3M sodium acetate and three volumes of chilled absolute ethanol was added and incubated overnight at 4°C. Precipitate was obtained by centrifugation at 12,000 rpm for 20 min. The precipitate was washed with 1 ml of 70% ethanol and centrifuged at 12,000 rpm for 10 min. The pellet was air dried and dissolved in 50 μl of nuclease free water and stored at −20°C for further use.

### PCR assay

*Cytochrome b* gene of *T. annulata* was targeted for its detection and identification. A primer set (Forward: 5’-ACT TTG GCC GTA ATG TTA AAC-3’/Reverse: 5’-CTC TGG ACC AAC TGT TTG G-3’) was used for amplification which was earlier reported by Bilgic *et al*. [6]. PCR assay was carried out in 200 μl PCR tubes using ABI thermocycler. Each 12.5 μl reaction mixture comprised 2 μl of template DNA, 6.25 μl Dream Taq Green PCR Master Mix (×2) (Fermentas), 0.5 μl of each cytob1 primer set (forward and reverse primer), and 3.25 μl nuclease free water. The PCR conditions includes initial denaturation at 95°C for 2 min; followed by 30 cycles of 95°C for 30 s (denaturation), 60°C for 30 s (annealing), and 72°C for 1min (extension); with a final extension step of 72°C for 10 min. 5 μl of amplified PCR product mixed with 6x loading dye was loaded for electrophoresis in 1.5% agarose gel along with 100 bp DNA ladder. The images were captured and documented using gel documentation system (Bio Rad., USA).

## Results

**Figure 1:**
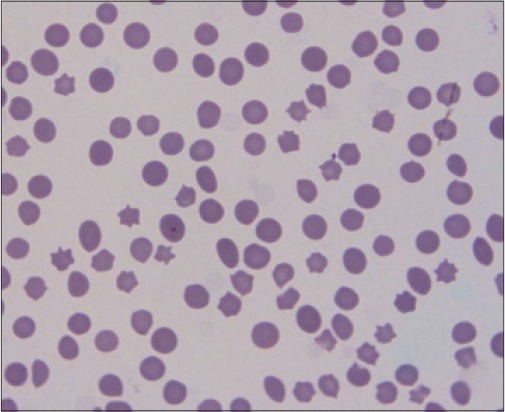
Signet ring intra erythrocytic piroplasm situated on or near the margin of the erythrocytes.

**Figure 2:**
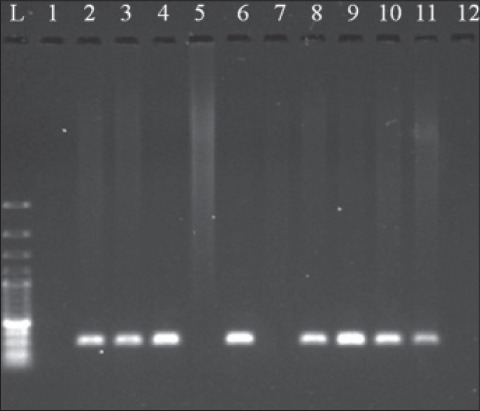
Gel electrophoresis results of polymerase chain reaction products showing specific amplification of 312 bp. L-Ladder 100bp; 1- Control negative; 2- Control positive; 3-12- Test samples; 5,7 and 12- Negative; 3,4,6,8,9,10,11-Positive.

**Figure 3:**
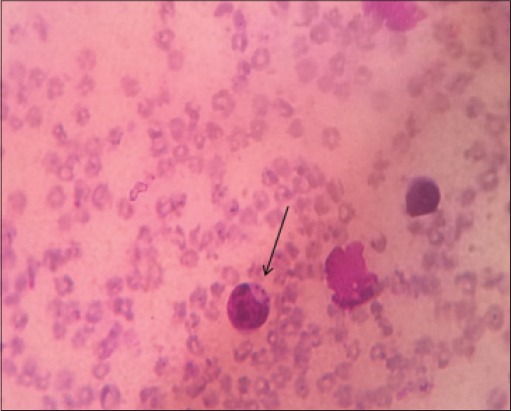
Koch’s blue bodies (macroschizont stage) in lymphocyte in Giemsa stained lymph node biopsy smear.

Out of 29 samples, 10 (34.48%) samples were found positive in Giemsa’s stained blood smears showing characteristic shaped piroplasm (Figure-1), whereas 18 (62.06%) were found positive for theileriosis by PCR assay (Table-1). Gel electrophoresis of PCR products showed specific amplicon of 312bp (Figure-2). The samples found negative with PCR assay were also found to be negative on blood smear examination. Koch’s blue bodies were seen in biopsy made from swollen lymph nodes of all four cows (Figure-3). On clinical examination, the increase in rectal temperature was seen in 5(17.24%) and swollen lymph nodes in 4 (13.79%) animals out of 29 screened. Five animals showing fever were diagnosed positive for theileriosis using microscopic examination as well as PCR assay. However, 13(72.22%) out of 18 animals diagnosed positive by PCR assay were asymptomatic carrier for theileriosis. Among 18 positive cases, 16 were adult whereas only 2 calves were found affected. PCR assay was negative on blood samples of cattle infected with *Babesia bigemina* and *Trypanosoma evansi*.

**Table-1:** Comparison of microscopy and PCR assay for detection of *T. annulata* in terms of prevalence.

Diagnostic test	Sample tested		Positive	Prevalence(%)

	Symptomatic animals	Asymptomatic animals		
Microscopy	5	24	10	34.48
PCR	5	24	18	62.06

*T. annulata=Theileria annulata*, PCR=Polymerase chain reaction

## Discussion

*T. annulata* is one of the most prevalent intra erythrocytic hemoprotozoan affecting crossbred cattle in India. Identification of carrier cattle is important as they are main source for the maintenance and perpetuation of *Theileria* in the environment and plays an important role in control program for theileriosis. Cattle become carriers when they survive the acute phase of the disease. After recovery, a low number of *T. annulata* piroplasms still remains in the body. In the most laboratories, detection of theileriosis is done by examining 50 microscopic fields for detection of parasites in blood smears which is a specific diagnosis but lacks sensitivity. In spite of the fact that method being cheaper, quicker, and easier to perform still requires high parasitemia, good smear preparation, proper staining and a well-trained microscopist. Although microscopic examination remains the convenient technique for day-to-day diagnosis of clinical cases, yet it is not able to detect carrier cattle due to the very low numbers (10^−3^× 5x 10^−1^) of infected erythrocytes [7].

PCR is now routinely used around the world for investigations of *Theileria* infections, particularly to determine carrier animals [6,8]. The forward and reverse primers used in this study were previously designed as a *T. annulata* species-specific primer based on *cytochrome b* genesequences for the detection of this parasite in the carrier animals [5].

In this study, the prevalence of theileriosis was found to be 62.06% using PCR assay, whereas only 34.48% using microscopy which clearly indicates that PCR assay is the more sensitive tool in diagnosing theileriosis. It has been revealed that microscopic examination is a less sensitive method which has also been reported by other workers [9-15]. However, both microscopy and PCR assay were found to be 100% specific in the diagnosis of theileriosis. PCR assay showed no cross-reactivity with *Babesia* sp. and *Trypanosoma* sp. and can be used specifically to diagnose theileriosis in herd and differentiate it from other hemoprotozoan diseases.

By relating clinical findings with results from conventional staining and PCR assay, it has been revealed that a large section of screened herd is asymptomatic carrier.

## Conclusions

From this study, it can be concluded that detection of asymptomatic carrier state is difficult to diagnose by conventional staining, whereas PCR assay can precisely determine the carrier state. Results received from PCR assay will certainly help in planning control programs.

## Authors’ Contributions

GC and NKR designed and planned the study. GC and NKR collected the samples and prepared the onsite smears slides. SM, AK, and GC performed biotechnological part of the study. NKR, TK, and RJ examined the stained smear. GC and AK drafted the manuscript. SM critically reviewed the manuscript. All authors read and approved the final manuscript. NKR finalized the manuscript and sent for publication.
